# The Effect of Acerola Intake on Metabolic and Immunological Parameters in Elite Athletes

**DOI:** 10.1007/s11130-025-01434-4

**Published:** 2025-11-21

**Authors:** Libor Vítek, Jana Hajšlová, Miloš Bátovský, Jana Woronyczová, Helena Posová, Miroslava Zelenková, Jaroslav Pilný, Marta Kalousová

**Affiliations:** 1 Institute of Medical Biochemistry and Laboratory Diagnostics, General University Hospital in Prague and 1 st Faculty of Medicine, Charles University, Prague, Czech Republic; 2 4th Department of Internal Medicine, General University Hospital in Prague and 1st Faculty of Medicine, Charles University, Prague, Czech Republic; 3 Department of Food Analysis and Nutrition, University of Chemistry and Technology, Prague, Czech Republic; 4 Slovak Army Sport Centre Dukla Banská Bystrica, Banská Bystrica, Slovak Republic; 5 Sports Research Institute of the Czech Armed Forces, Prague, Czech Republic; 6 Institute of Anatomy, Faculty of Medicine in Hradec Kralove, Charles University, Hradec Kralove, Czech Republic

**Keywords:** Acerola, Immune system, Inflammation, Metabolic flexibility, Oxidative stress, Sport

## Abstract

Acerola is an edible tropical fruit known for its high antioxidant activity. It is recommended as a superfood to improve general health. However, its role in elite sport has not been studied in detail so far. Therefore, the objective of the study was to assess the effect of regular use of acerola on parameters of the immune system, oxidative stress, and metabolism in elite athletes. The study was carried out in 22 elite endurance athletes (mean age = 24.4 ± 4 years; M:F ratio = 1.75), who were supplemented with acerola pulp at a dose of 300 g/day for 3 weeks. Detailed laboratory studies, including analyses of biochemical, hematological, and immunological parameters, were carried out in all subjects before and after acerola supplementation. Acerola supplementation did not change any tested parameters of oxidative stress but decreased serum immunoglobulin concentrations and some inflammatory markers. Simultaneously, acerola significantly decreased serum glucose, urea, and liver enzymes ALT and AST, suggesting its role in modulating metabolic flexibility. Acerola supplementation appears to play a beneficial role in elite endurance sports by improving low-grade inflammatory status and metabolic flexibility. Long-term, larger studies are needed to confirm current data and the possible impact on sports performance, as well as to assess any side effects of chronic use.

## Introduction

Acerola is the edible fruit of the tropical shrub *Malpighia emarginata DC (alias M. glabra* L.), a native of Central America, which belongs to the *Malpighiaceae* family. It is important for human nutrition due to its potent antioxidant activity, mainly due to its high content of L-ascorbic acid (vitamin C), but also other antioxidants such as carotenoids, flavonoids, and anthocyanins [1]. The fruit contains ascorbic acid in an amount approximately 50 to 100 times higher than that of orange or lemon, therefore, it is considered an important ¨superfruit/superfood¨, which should become a regular component of the human diet [2]. The role of antioxidants in elite sport has been a subject of vivid discussion recently, with both positive as well as warning conclusions [3, 4].

Sports foods and supplements can play an important role in high performance athletes´ sports nutrition plans. In fact, the prevalence of the use of dietary supplements among athletes has been reported between 40 and 100%, depending on several factors, including the level of competition, the type of sport, and the definition of dietary supplement use [5]. Although thousands of such supplements are on the market, only a few of them can really improve athletic performance. Because of that, the Australian Institute of Sport (AIS) has developed a Sports Supplement Framework with the ABCD Classification system to recognize and differentiate sports supplements according to their efficacy and health safety [6]. Interestingly, fruit-derived polyphenols and vitamin C, abundantly contained in acerola, are classified in Group B, with the level of evidence emerging scientific support, which deserves of further research [6].

It should also be noted that there has currently been a decline in the use of synthetic dietary supplements in sport, which are substituted for a food-first approach/strategy [7]. In fact, recent data suggest that chronic intake of high doses of antioxidants such as ascorbate can hinder training adaptations [8], delay post-exercise recovery, and even increase oxidative stress (for a review, see [4]). However, when vitamin C is taken from natural food sources, performance is believed to improve [9]. Acerola is also rich in many bioactive metabolites, including polyphenolic compounds [1], whose intake is suggested, according to a recent systematic review, to promote sport performance [10]. Based on the high content of bioactive compounds, acerola could enhance athletic performance, but scientific evidence for this suggested beneficial effect is lacking. There are at least three possible explanations, why acerola could be beneficial in sports performance: (1) to improve increased oxidative stress defense [3], (2) to decline low-grade chronic inflammation (LGCI) [11], and (3) to modulate metabolic flexibility and homeostasis of energy sources [12].

Therefore, the objective of our study was to assess the role of regular use of acerola on the parameters of the immune system, oxidative stress, and metabolism in a group of elite athletes to evaluate the nutraceutical potential of acerola in elite athletes on their health status.

## Materials and Methods

### Subjects

In our pilot study, a total of 22 consecutive elite endurance athletes (mean age = 24.4 ± 4 years; M:F ratio = 1.75) were recruited from the Dukla Banská Bystrica Slovak Army Sports Center. The sport disciplines included biathlon, cross-country skiing, cycling, and long athletic runs (5,000 and 10,000 m). The main exclusion criterion was any acute or subacute illness. The athletes were supplemented with acerola pulp in a dose of 300 g/day in three daily doses for three weeks (Acerola fruit pulp, Frutamil, Brazil). Laboratory analyses were carried out in all subjects before and after acerola supplementation. All athletes were instructed to stop using any other supplements containing vitamins, trace elements, or flavonoids for three weeks prior to starting the study. They were also instructed to keep their training regimens, diet, and other lifestyle conditions exactly the same as before starting acerola supplementation.

The whole study was carried out in accordance with the Helsinki Declaration of 1975, as revised in 1983. All participants provided their informed consent. The study was approved by the Ethics Committee of the CASRI (Sports Research Institute of the Czech Armed Forces) (Nos. 6/1–6/8 2019). Participant confidentiality was kept according to General Data Protection Regulation (GDPR) by European Union (2018). Participants were instructed to record all possible adverse events and report them immediately to the study investigators.

### Blood Collection and Laboratory Analyses

In elite athletes, a venous blood samples (2 × 7 mL) were collected in the morning in the fasting state and analyzed for standard biochemical and hematological parameters using routine clinical assays on automatic analyzers (Cobas R8000 Modular analyzer, Roche Diagnostics GmbH, Mannheim, Germany; and Sysmex XN-550, Sysmex, Kobe, Japan, respectively).

Blood was centrifuged (1,500 *g* for 15 min) and serum was immediately stored at − 80 °C for further antioxidant analyses performed within a month after blood sampling.

### Determination of Total Antioxidant Status and Glutathione Reductase Activity

Total antioxidant status (TAS, expressed in mmol/L) and glutathione reductase activity (GR, expressed in U/L) were determined in athletes using TAS and GLUT RED spectrophotometric kits, respectively, according to the manufacturer’s instructions (Randox Laboratories Ltd., UK) on automatic analyzer (Chemistry Analyzer BS-240, Mindray Bio-Medical Electronics, China).

### Immunological Analyses

The concentration of mannose binding lectin (MBL) was measured using a commercially available ELISA kit (R&D Systems, MN, USA) according to the manufacturer’s instructions, with reference values between 103 and 3308 ng/mL. Nephelometric measurements to quantitatively determine serum concentrations of C1q, C3, and C4 complement components were performed with a Siemens BN-II nephelometer (Siemens Healthineers, Germany). The reference values for C3 and C4 were 0.9–1.8 mg/L and 0.1–0.4 mg/L, respectively.

Inflammatory cytokines were analyzed using the MAGPIX system (Luminex Corp., Austin, TX, USA) using the MILLIPLEX MAP kit (Merck KGaA, Darmstadt, Germany) according to the manucafturer´s instructions and using MILLIPLEX^®^ Analyst 5.1 software (Merck KGaA, Darmstadt, Germany).

White blood cell subpopulations were determined by flow cytometry. Fifty µl of whole blood was taken from sodium-heparin Vacutainer tubes and the following stained antibodies (Beckman Coulter, CA, USA) were used: CD45 KrO (Krome Orange), CD3 AA750 (APC-Alexa Fluor 750), CD4 PB (Pacific Blue), CD3FITC/CD16 + 56PE, CD8 AA700 (APC-Alexa Fluor 700), CD27 PB, CD127 FITC, CD25 ECD R (Phycoerythrin-Texas Red^®^-X) and fully processed in Beckman Coulter Tq-Prep using the Immunoprep Reagent System. Subsequently, the samples were immediately measured using a flow cytometer (Beckman Coulter Navios Ex System).

### Acerola Analysis Using U-HPLC-HRMS/MS Metabolomic Fingerprinting

For qualitative analysis of polyphenolic compounds in acerola pulp extract, a metabolomic fingerprint strategy based on ultra-high performance liquid chromatography coupled with tandem high-resolution mass spectrometry (U-HPLC-HRMS/MS, TripleTOF 6600, SCIEX, MA, USA) was used for this approach. The data obtained were analyzed using PeakView 2.0 software. The target screening of potentially present polyphenols was performed against the in-house spectral library prepared on the basis of literature data. Vitamin C and E concentrations were quantitatively analyzed using the same U-HPLC-HRMS/MS apparatus.

### Statistical Analyses

Data are expressed as mean ± SD, or as median and interquartile range when the data were not distributed normally. Based on data normality, the paired T test or the Signed Rank test were used to compare laboratory parameters in individual subjects, who themselves served their own control. All analyses were performed with the alpha set to 0.05. Statistics were calculated using SigmaPlot v. 14.5 (Systat Software, Inc. CA, USA).

## Results and Discussion

### Analysis of Bioactive Substances in Acerola Pulp

The acerola pulp used in the study contained high amounts of vitamin C (873 ± 70 mg/100 g, Table 1) and was rich in flavonoids. In fact, quercitrin and aceronidin were the most abundant polyphenolic compounds, other polyphenols were present in lower, but still biologically significant amounts (Table 2). In addition to these bioactive metabolites, the presence of various carotenoids (some of them vitamin A provitamins) was detected.

**Table 1:** Antioxidant vitamins content in the acerola pulp

	Concentration (mg/100 g)
Vitamin C	873
Vitamin E	1.4

**Table 2:** Profile of polyphenolic compounds in the acerola pulp

Polyphenolic compounds	Elemental formula	Theoretical m/z value	Measured m/z value	Signal intensity
Quercetin 3-O-rhamnoside (quercitrin)	C_21_H_20_O_11_	447.09329	447.09368	297,610
Leucocyanidin 3-O-β-D-glucoside (aceronidin)	C_21_H_22_O_11_	449.10894	449.10933	241,871
Quercetin 3-glucoside (isoquercitrin)	C_21_H_20_O_12_	463.08820	463.08824	98,802
Quercetin	C_15_H_10_O_7_	301.03538	301.03541	63,290
Cyanidin 3-rhamnoside	C_21_H_20_O_10_	431.09837	431.09796	38,111
Kaempferol	C_15_H_10_O_6_	285.04046	285.04051	22,087
Quercetin 3-glucoside-7-O-rhamnoside	C_27_H_30_O_16_	609.14611	609.14581	21,653
Pelargonidin 3-rhamnoside	C_21_H_20_O_9_	415.10346	415.10309	21,435
Epicatechin	C_15_H_14_O_6_	289.07176	289.07186	13,930
Isorhamnetin 3-O-glucoside	C_22_H_22_O_12_	477.10385	477.10406	8463
Naringenin/Pinobanksin	C_15_H_12_O_5_	271.06120	271.06135	7484
Isorhamnetin	C_16_H_12_O_7_	315.05103	315.05121	5939

Data expressed as relative proportion of individual polyphenolic compounds

Polyphenolic compounds in acerola pulp extract were analyzed qualitatively using a metabolomic fingerprint strategy based on U-HPLC-HRMS/MS. The target screening of potentially present polyphenols was performed against the in-house spectral library

### The Effect of Acerola Supplementation on Hematological, Immune System, Oxidative Stress, and Metabolic Markers

Acerola supplementation led to a slight but significant decrease in quantitative markers of red blood cells, as well as neutrophil and white blood counts (Table 3). Lymphocyte counts were comparable between preexposure and postexposure groups, similarly to individual lymphocyte subpopulations (Table 3).

**Table 3:** The effect of acerola intake on hematological, immune system and oxidative stress parameters

Parameter (reference range, units)	Pre-acerola	Post-acerola	*P*-value
Hematological markers			
RBC (3.8–5.2 × 10^12^/L)	5 (4.5–5.2)	4.8 (4.5–5.2)	**0.02**
Hemoglobin (120–175 g/L)	149.3 ± 13.1	144.5 ± 10.9	**0.001**
Hematocrit	0.447 ± 0.04	0.426 ± 0.03	**< 0.001**
WBC (4–10 × 10^9^/L)	5.79 (4.7–7.7)	4.97 (3.9–6.5)	0.16
Platelets (150–400 × 10^9^/L)	217 (209–250)	197 (176–233)	**< 0.001**
Neutrophils (2–7 × 10^9^/L)	3.1 (2.3–4.2)	2.4 (1.8–3.7)	**0.001**
Lymphocytes (2–7 × 10^9^/L)	2 ± 0.5	2.1 ± 0.5	0.49
CD4^+^/CD8^+^ (IRI 1–3/L)	1.79 ± 0.5	1.85 ± 0.6	0.21
CD3^+^CD4^+^ (0.3–2.8 × 10^9^/L)	0.79 (0.7–0.96)	0.82 (0.66 − 0.11)	0.78
CD3^−^CD16^+^CD56^+^ (0.05–1.05 × 10^9^/L)	0.31 ± 0.16	0.33 ± 0.17	0.48
CD4^+^CD25^+^CD127^−^ (4.7–10.5% of CD4)	4 (3.4–4.3)	3.9 (3.5–4.6)	0.72
Inflammatory markers			
IgG (7–16 g/L)	10.94 ± 2.2	9.72 ± 2	**< 0.001**
IgG1 (4.9–11.4 g/L)	6.94 ± 1.3	6.17 ± 1.3	**0.001**
IgG2 (1.5–6.4 g/L)	3.2 ± 1.1	2.92 ± 1	**< 0.001**
IgG3 (0.2–1.1 g/L)	0.4 (0.2–0.4)	0.3 (0.2–0.4)	**0.008**
IgG4 (0.08–1.4 g/L)	1 (0.4–1.3)	0.9 (0.4–1.2)	**0.02**
IgA (0.7–4 g/L)	1.7 (1.2–2.4)	1.6 (1.1–2.1)	**< 0.001**
IgM (0.4–2.3 g/L)	1 (0.8–1.2)	1 (0.8–1.3)	NS
IgE (0–100 g/L)	30 (3.8–79.5)	36.1 (6.5–89.8)	**0.05**
C1q (146–322 mg/L)	144 (132–162)	126 (115–140)	**< 0.001**
C3 (0.9–1.8 g/L)	0.886 ± 0.14	0.913 ± 0.16	NS
C4 (0.1–0.4 g/L)	0.197 ± 0.06	0.202 ± 0.07	NS
MBL (103–3308 µg/L)	698 (364–918)	715 (301–918)	0.18
Serum amyloid A (0–6.8.8 mg/L)	4 (3.4–7.8)	3.4 (3.4–4.9)	NS
Ferritin (10–322 (µg/L)	81.5 ± 48	76.8 ± 51	NS
CRP (0–10 mg/L)	1 (1–8)	1 (1–6)	0.84
TNF-α (ng/L)	15.4 (9.9–20.4)	14.3 (11.2–27)	0.67
IL-6 (ng/L)	85.6 ± 148	89.8 ± 141	0.68
IFNγ (ng/L)	28.6 (9.1–73)	26 (8.2–114)	0.17
IL-17 A (ng/L)	20.9 (12.3–85.5)	29.6 (10.5–53.3)	0.14
sCD40L (ng/L)	7152 ± 2930	6841 ± 2802	0.19
IL-8 (ng/L)	14 (6.5–25)	18.7 (9.1–27)	0.40
Oxidative stress markers			
TAS (1.3–1.77 mmol/L)	1.38 ± 0.1	1.4 ± 0.1	0.56
GR reductase (33–73 U/L)	61.5 ± 10.6	64.2 ± 7.8	0.31

Data stated as mean ± SD or median (IQ range) when non-normally distributed

*C3* C3 complement component,* C4* C4 complement component, *CRP* C-reactive protein, *GSH* glutathione, *GR* glutathione reductase, *IFN *interferon, *IL* interleukin, *IRI* immunoregulatory index, *MBL* mannose binding lectin, *RBC* red blood cells, *sCD40L* soluble CD40 ligand, *TAS* total antioxidant status, TNF-α, tumor necrosis factor-α; *WBC *white blood cells

Subjects supplemented with acerola had significantly lower concentrations of IgG and IgA immunoglobulins, including all IgG subgroups (Table 3). They also had lower concentrations of the complement component C1q, a known inflammatory marker (Table 3) [13].

No differences in GR activity and TAS were detected in the subjects examined (Table 3).

Interestingly, 3-week exposure to acerola led to significant improvements in the concentrations of metabolic markers, such as glucose (4.8 vs. 4.6 mmol/L, *p* = 0.001), urea (6.3 vs. 5.8 mmol/L, *p* = 0.016), as well as the activities of the liver enzymes ALT (0.48 vs. 0.41 µkat/L) and AST (0.51 vs. 0.40 µkat/L) (Table 4).

**Table 4:** The effect of acerola intake on serum metabolic parameters

Parameter (units, reference range)	Pre-acerola	Post-acerola	*P*-value
Glucose (3.9–5.6 mmol/L)	4.8 (4.7–5.1)	4.6 (4.3–4.7)	0.001
Urea (2–8 mmol/L)	6.3 (5.2–7.2)	5.8 (4.5–6.7)	0.016
Bilirubin (3.8–27.5 µmol/L)	12.5 (9.6–16.6)	12.3 (9.8–17.8)	0.63
ALT (0.1–0.78 µkat/L)	0.48 ± 0.15	0.41 ± 0.13	0.005
AST (0.1–0.72 µkat/L)	0.51 ± 0.15	0.40 ± 0.14	< 0.001
Total cholesterol (2.9–5.0.9.0 mmol/L)	4.64 ± 1	4.61 ± 0.8	0.82

Data stated as mean ± SD or median (IQ range) when non-normally distributed

Acerola is a tropical fruit known for its high antioxidant activity, which is believed to be due not only to its high content of vitamin C, but also to other antioxidants such as carotenoids, flavonoids, and anthocyanins [1]. In fact, a high content of vitamin C was detected in the analyzed acerola pulp (Table 1), overcoming those of commonly recommended vitamin C dietary sources. However, we did not observe a significant improvement in two markers of oxidative stress (TAS and GR) analyzed in our athletes supplemented with acerola, which may be due to lack of the lack of any effect on redox status, or due to non-specificity of the assays used [14]. On the other hand, specific markers of immune system status and metabolic parameters improved significantly in athletes supplemented with acerola.

LGCI affects sports performance. While moderate physical activity is associated with decreased production of pro-inflammatory signals, intense physical training predisposes to the LGCI status [11, 15]. This can result in higher susceptibility to infections [16], decreased performance [17], increased risk of injuries, and longer recovery [18]. LGCI is also important in the pathogenesis of overreaching and overtraining syndromes [11]. LGCI negatively affects cardiovascular functions and is also associated with a tendency to metabolic acidosis [11]. Since pharmacological options are limited in elite sports, there is a search for nutraceutical approaches to limit LGCI in athletes. Based on our data and also on published reports, it appears that acerola supplements can ameliorate LGCI in elite athletes with all immunological, musculoskeletal and metabolic consequences. In fact, acerola supplementation led to a decrease in neutrophil counts, a phenomenon likely attributable to the anti-inflammatory action of acerola [19], as higher neutrophil counts are indeed associated with chronic inflammation [20]. This is supported by a significantly lower platelet count in athletes supplemented with acerola (Table 3), since platelets play an integral role in the inflammatory response and immune regulation [21]. Athletes supplemented with acerola also had lower serum concentrations of IgG (including all IgG subclasses) and IgA, responsible for adaptive immunity and being markers of LGCI [22].

Although most of the analyzed acute phase proteins did not change after exposure to acerola, a significant decrease was observed in the complement component C1q in supplemented athletes. C1q belongs to positive inflammatory markers [13] that increase in inflammatory conditions, particularly respiratory infections. It serves as a marker of the status of the complement system, as well as of the activity of the adaptive immune system [23]. Interestingly, acerola supplementation in our athletes led to a decrease in serum C1q concentrations that supports its possible anti-inflammatory action (Table 3).

Surprisingly, there is a scarcity in clinical data assessing possible beneficial effects on metabolic diseases such as diabetes, metabolic syndrome, or liver steatosis/steatohepatitis.

The hepatoprotective effect of acerola supplementation was observed in animal models of liver injury [24, 25]. In addition, the protective effect of acerola against liver steatosis was also demonstrated experimentally [26], but so far no clinical studies on liver protection have been carried out. Furthermore, hypoglycemic and hypocholesterolemic effects were reported in experimental in vitro as well as animal studies [27, 28], but again clinical evidence from human studies is lacking. In our study on elite athletes, serum liver enzyme activities, as well as serum glucose and urea concentrations improved (Table 3) suggesting that acerola supplementation can improve metabolic flexibility, positively associated with athletic performance [12].

### Can Regular Acerola Intake Have any Ergogenic effect?

On the basis of our data and reports discussed above, it appears that acerola can affect at least several factors contributing to better physical performance:

- Acerola improves glucose stability. Low glucose variability is not only important for the prevention of diabetes [29], but is believed to also significantly affect importantly physical performance in elite athletes [30, 31].
- Acerola improves the basal activity of liver enzymes. Although no studies have been reported on the possible link between liver enzyme activities and sport performance, liver enzymes are important predictors of morbidity and mortality, and even variability in their activities is an unfavorable predictive factor [32, 33].
- Acerola improves immunological status and decreases LGCI. This is suggested by the results of our study, but also addressed in detail in a recent paper by Olędzki and Harasym (for a review, see [34]).
- Effects mediated by specific substances found in acerola. Interestingly, quercetin, found in high amounts in tested acerola (Table 2), was found in a recent systematic review to have anti-inflammatory and performance-enhancing activities [35].

Our study has several limitations. The cohort size was small; a larger sample size would certainly improve the reliability and generalizability of the findings. In addition, due to the small sample size, it was not possible to assess possible differences in responses based on subgroups, such as gender or training status. Finally, the three-week duration of acerola supplementation is certainly not sufficient to observe prolonged effects on metabolic and immunological parameters.

## Conclusions

Acerola supplementation appears to play a beneficial role in elite endurance sports by improving low-grade inflammatory status and metabolic flexibility. Based on these results, it appears that acerola supplements could be recommended for athletes as well as the general population. Long-term larger studies are needed to confirm current data and the possible impact on sports performance, as well as to assess any side effects of chronic use.

## Data Availability

The data that support the findings of this study are available from the corresponding author upon reasonable request.

## Abbreviations

ALT: alanine aminotransferase
AST: aspartate aminotransferase
GR: glutathione reductase activity
LGCI: low-grade chronic inflammation
MBL: mannose binding lectin
TAS: total antioxidant status
U-HPLC-HRMS/MS: ultra-high performance liquid chromatography coupled with tandem high-resolution mass spectrometry
